# Variation in the tonoplast cadmium transporter heavy metal ATPase 3 (*HMA3*) homolog gene in *Aegilops tauschii*

**DOI:** 10.1371/journal.pone.0279707

**Published:** 2023-03-03

**Authors:** Shengke Li, Xiao Li, Shijie Li, Yu’e Liu, Tianqing Zang, Ming Hao, Lianquan Zhang, Lin Huang, Bo Jiang, Zhongwei Yuan, Xuejiao Chen, Xue Chen, Dengcai Liu, Shunzong Ning

**Affiliations:** 1 State Key Laboratory of Crop Gene Exploration and Utilization in Southwest China, Sichuan Agricultural University, Chengdu, Sichuan, China; 2 Triticeae Research Institute, Sichuan Agricultural University, Chengdu, Sichuan, China; Saint Mary’s University, CANADA

## Abstract

The functionality of *HMA3* is a key determinant controlling Cd accumulation in the shoots and grains of plants. Wild relatives of modern crop plants can serve as sources of valuable genetic variation for various traits. Here, resequencing of *HMA3* homoeologous genes from *Aegilops tauschii* (the donor of the wheat D genome) was carried out to identify natural variation at both the nucleotide and polypeptide levels. *HMA3* homoeologs are highly conserved, and 10 haplotypes were revealed based on 19 single nucleotide polymorphisms (eight induced single amino acid residue substitutions, including 2 altered amino acids in transmembrane domains) in 80 widely distributed *Ae*. *tauschii* accessions. The results provide genetic resources for low/no Cd concentration wheat improvement.

## Introduction

The low cadmium (Cd) concentration trait in bread wheat (*Triticum aestivum* L., AABBDD, 2n = 6x = 42) grains is important in terms of food safety because bread wheat is a crucial source of calories, accounting for 20% of calories for human consumption and feeding more than 35% of the world’s population [1]. Cadmium can accumulate in the human body over time as a result of the ingestion of food containing Cd, leading to a risk of chronic toxicity with excessive intake [2]. Furthermore, Cd is not phytotoxic at the low concentrations that are of concern for human health [3]. Therefore, reducing Cd accumulation in wheat grains is essential for food safety and human health issue.

The tonoplast transporter HMA3 (P_1B_-type of heavy metal ATPases 3) plays an important role in the transport and homeostasis of metals in plants [4]. Loss of function of *HMA3* leads to decreased Cd sequestration in the roots but greatly increased translocation of Cd from roots to shoots in *Arabidopsis thaliana* and rice [5–8]. In contrast, overexpression of *OsHMA3* increases Cd sequestration in the roots and Cd tolerance, and markedly decreases Cd translocation to the shoots and Cd concentration in the grains (decreased Cd concentration by 94–98%) [9, 10]. Overexpression of rice *OsHMA3* in wheat also greatly decreases Cd translocation from the roots to the shoots and Cd accumulation in wheat grains [11]. Recently, *TdHMA3-B1* (*Cdu-B1*) was identified as a QTL located on the long arm of chromosome 5B that explains >80% of the phenotypic variation in grain Cd concentration in tetraploid durum wheat (*T*. *durum*, AABB, 2n = 4x = 28) [12–16]. Cd associated SNP markers on 5AL were identified in a region homoeologous to the *TdHMA3-B1* locus on 5BL in durum wheat, which explained 12%-19% of variation in grain Cd concentration depending on the environment in bread wheat [17]. A 17-bp duplication in the first exon that creates two alternative alleles, *TdHMA3-B1a* (the functional allele being able to transport Cd into the vacuoles with low grain Cd) and *TdHMA3-B1b* (the nonfunctional allele being inactive with high grain Cd), in durum wheat [16]. In addition, the high-cadmium allele is widespread in durum wheat cultivars but undetected in wild emmer accessions, the frequency of the high-cadmium allele are increased from domesticated emmer accessions to modern durum wheat [16]. These studies indicate that the functionality of *HMA3* is a key determinant controlling Cd accumulation in the shoots and grains of plants and that wild relatives of modern crop plants can serve as sources of valuable genetic diversity to develop low/no Cd concentration cultivars.

*Aegilops tauschii* (DD, 2n = 2x = 14), a wild relative of wheat, is the D genome progenitor of hexaploid wheat [18–21]. It has been shown to be an effective medium for transferring valuable genetic variation from *Ae*. *tauschii* to common wheat [22–24]. The addition of the D genome changed wheat from tetraploid to hexaploid. Indeed, hexaploid wheat became a major food crop worldwide only after the addition of the D genome [25]. It can be seen that their ability to spread widely may be related to the great adaptability brought by the D genome, which also explains why they have many agronomic valuable characteristics, including tolerance to drought [26], salt [27], low phosphorus [28] and extreme environments. Besides that, The Cd-tolerant *Ae*. *tauschii* accessions were indentified by Genome-wide association analysis, which may be used as germplasm resources of wheat breeding for no or low Cd content [29]. Here, we report a survey of the natural variation in potential *Ae*. *tauschii* germplasm for *HMA3* homoeologous sequences across a diverse geographical distribution and provide genetic resources for low/no Cd concentration wheat improvement.

## Materials and methods

### Plant materials

The *Ae*. *tauschii* germplasm panel consisted of 80 accessions (Table 1). The materials were planted at the Wenjiang Experimental Station of Sichuan Agricultural University in Chengdu, China. All materials were stored at the Triticeae Research Institute, Sichuan Agricultural University.

**Table 1:** Information on *Ae*. *tauschii* samples used in this study.

Lines	Origin	Latitude	Longitude	Sublineage	Taxon	Haplotype	GenBank Accession No.
PI603241	Azerbaijan	40.53	48.92	2W	ssp. *strangulata*	Hap-D1	OL739674
CIae28	Iran	37.98	45.02	1W	ssp. *tauschii*	Hap-D1	OL739674
AS68	Iran	NA	NA	1E	ssp. *tauschii*	Hap-D1	OL739674
IG48556	Tajikistan	39.47	67.83	1E	ssp. *tauschii*	Hap-D1	OL739674
PI554315	Turkey	38.42	43.30	1W	ssp. *tauschii*	Hap-D1	OL739674
PI554309	Turkey	38.46	42.45	1W	ssp. *tauschii*	Hap-D1	OL739674
PI554312	Turkey	38.42	43.30	1W	ssp. *tauschii*	Hap-D1	OL739674
PI560753	Turkey	37.48	43.53	1W	ssp. *tauschii*	Hap-D1	OL739674
PI603220	Western Asia	NA	NA	1E	ssp. *tauschii*	Hap-D1	OL739674
CIae71	NA	NA	NA	1E	ssp. *tauschii*	Hap-D1	OL739674
AS84	NA	NA	NA	NA	ssp. *tauschii*	Hap-D1	OL739674
PI499263	NA	NA	NA	NA	ssp. *tauschii*	Hap-D1	OL739674
KU2615	NA	NA	NA	NA	ssp. *tauschii*	Hap-D1	OL739674
DV148	NA	NA	NA	NA	ssp. *tauschii*	Hap-D1	OL739674
AE251	NA	NA	NA	NA	ssp. *tauschii*	Hap-D1	OL739674
KU2082	NA	NA	NA	NA	ssp. *strangulata*	Hap-D1	OL739674
DV288	Afghanistan	37.23	70.29	NA	ssp. *tauschii*	Hap-D10	OL739683
DV306	Azerbaijan	40.58	48.73	NA	ssp. *strangulata*	Hap-D10	OL739683
PI603240	Azerbaijan	40.63	48.62	2W	ssp. *strangulata*	Hap-D10	OL739683
CIae15	Iran	35.76	52.78	2W	ssp. *strangulata*	Hap-D10	OL739683
PI603226	Iran	36.58	53.50	2E	ssp. *strangulata*	Hap-D10	OL739683
PI603250	Iran	36.67	53.58	2E	ssp. *strangulata*	Hap-D10	OL739683
PI554311	Turkey	38.42	43.30	1W	ssp. *tauschii*	Hap-D10	OL739683
IG123880	Uzbekistan	41.24	71.65	1E	ssp. *tauschii*	Hap-D10	OL739683
CIae72	NA	NA	NA	2E	ssp. *strangulata*	Hap-D10	OL739683
DV634	NA	NA	NA	2E	ssp. *strangulata*	Hap-D10	OL739683
AL370	Iran	38.24	48.28	2E	ssp. *strangulata*	Hap-D10	OL739683
KU2078	Iran	36.85	54.53	2E	ssp. *strangulata*	Hap-D10	OL739683
AE1602	NA	NA	NA	NA	ssp. *strangulata*	Hap-D10	OL739683
AE425	NA	NA	NA	NA	ssp. *strangulata*	Hap-D10	OL739683
KU2161	NA	NA	NA	NA	ssp. *strangulata*	Hap-D10	OL739683
DV140	Afghanistan	32.38	67.30	NA	ssp. *tauschii*	Hap-D2	OL739675
PI431601	Azerbaijan	40.50	47.00	2W	ssp. *strangulata*	Hap-D2	OL739675
PI486273	Turkey	40.15	43.37	1W	ssp. *tauschii*	Hap-D2	OL739675
PI511365	NA	NA	NA	NA	ssp. *tauschii*	Hap-D2	OL739675
PI560534	Turkey	37.58	43.73	1W	ssp. *tauschii*	Hap-D3	OL739676
PI486271	Turkey	38.92	43.60	1W	ssp. *tauschii*	Hap-D3	OL739676
PI220642	Afghanistan	35.72	64.90	1E	ssp. *tauschii*	Hap-D4	OL739677
PI511367	Afghanistan	34.53	69.18	1E	ssp. *tauschii*	Hap-D4	OL739677
DV189	Afghanistan	35.11	63.43	NA	ssp. *tauschii*	Hap-D4	OL739677
DV169	Afghanistan	35.99	64.87	NA	ssp. *tauschii*	Hap-D4	OL739677
DV185	Afghanistan	35.74	63.73	NA	ssp. *tauschii*	Hap-D4	OL739677
DV327	Armenia/Georgia	41.76	44.76	NA	ssp. *tauschii*	Hap-D4	OL739677
DV328	Georgia	42.02	44.31	NA	ssp. *tauschii*	Hap-D4	OL739677
CIae14	Iran	36.80	55.10	1E	ssp. *tauschii*	Hap-D4	OL739677
IG47234	Pakistan	30.50	67.00	1E	ssp. *tauschii*	Hap-D4	OL739677
AL7/79	Turkmenistan	39.21	62.25	1E	ssp. *tauschii*	Hap-D4	OL739677
IG48544	Uzbekistan	40.08	67.58	1E	ssp. *tauschii*	Hap-D4	OL739677
IG48570	Uzbekistan	40.88	71.10	1E	ssp. *tauschii*	Hap-D4	OL739677
IG47232	Pakistan	30.38	67.00	1E	ssp. *tauschii*	Hap-D4	OL739677
IG46670	Pakistan	29.93	66.62	1E	ssp. *tauschii*	Hap-D4	OL739677
IG48567	Uzbekistan	40.57	71.70	1E	ssp. *tauschii*	Hap-D4	OL739677
DV326	Armenia/Georgia	41.76	44.76	NA	ssp. *tauschii*	Hap-D4	OL739677
AE964	NA	NA	NA	NA	ssp. *tauschii*	Hap-D4	OL739677
AE841	NA	NA	NA	NA	ssp. *tauschii*	Hap-D4	OL739677
CIae12	Iran	36.70	55.12	2E	ssp. *strangulata*	Hap-D5	OL739678
PI560756	Turkey	37.70	43.97	1W	ssp. *tauschii*	Hap-D5	OL739678
KU2094	Iran	36.59	52.09	2E	ssp. *strangulata*	Hap-D5	OL739678
AL8/78	Armenia	40.30	44.66	2W	ssp. *strangulata*	Hap-D6	OL739679
DV2917	Azerbaijan	38.97	48.34	NA	ssp. *strangulata*	Hap-D6	OL739679
CIae8	Iran	36.67	53.40	2E	ssp. *strangulata*	Hap-D6	OL739679
PI542277	Turkey	NA	NA	2W	ssp. *strangulata*	Hap-D6	OL739679
PI486267	Turkey	37.27	44.55	2W	ssp. *strangulata*	Hap-D6	OL739679
PI574469	India	NA	NA	1E	ssp. *tauschii*	Hap-D6	OL739679
AE224	NA	NA	NA	NA	ssp. *strangulata*	Hap-D6	OL739679
KU2126	NA	NA	NA	NA	ssp. *strangulata*	Hap-D6	OL739679
DV331	Afghanistan	34.59	68.95	NA	ssp. *tauschii*	Hap-D7	OL739680
PI428564	Azerbaijan	40.50	47.00	2W	ssp. *strangulata*	Hap-D7	OL739680
PI603242	Turkmenistan	38.48	56.30	2E	ssp. *strangulata*	Hap-D7	OL739680
PI554317	Turkey	38.42	43.30	1W	ssp. *tauschii*	Hap-D7	OL739680
PI603230	Azerbaijan	40.50	47.00	2E	ssp. *strangulata*	Hap-D8	OL739681
PI603253	Iran	36.88	50.69	2E	ssp. *strangulata*	Hap-D8	OL739681
PI511381	Iran	36.67	53.58	2E	ssp. *strangulata*	Hap-D8	OL739681
DV198	Iran	36.82	54.29	NA	ssp. *strangulata*	Hap-D8	OL739681
K901/75	NA	NA	NA	2E	ssp. *strangulata*	Hap-D8	OL739681
AS2406	Iran	NA	NA	1E	ssp. *tauschii*	Hap-D8	OL739681
AE430	NA	NA	NA	NA	ssp. *strangulata*	Hap-D8	OL739681
AE1548	NA	NA	NA	NA	ssp. *strangulata*	Hap-D8	OL739681
PI317394	Afghanistan	34.95	63.22	1E	ssp. *tauschii*	Hap-D9	OL739682
PI349037	Azerbaijan	40.50	47.00	2E	ssp. *strangulata*	Hap-D9	OL739682

Accessions prefixed with either PI and CIae were provided by the USDA-ARS, those with KU by the Japanese National BioResource Project (NBRP), those with IG by the International Center for Agricultural Research in Ardid Areas (ICARDA), those with TA by Kansas State University, those with either K, AL and RL by UC Davis, and those with AS by Sichuan Agricultural University Triticeae Research Institute. Genotype (Sublineage) were previously published [30].

### PCR primer design, amplification and amplicon sequencing

Genomic DNA from plant materials was extracted from young leaves using a plant genomic DNA kit (Tiangen Biotech (Beijing) Co., Ltd.). The PCR primers were designed according to the sequences of *TaHMA3-D1* (KF683298.1) and its homologous sequence in the EnsemblPlants database (http://plants.ensembl.org/Aegilops_tauschii/Info/Index) using DNAMAN version 6.0 (Lynnon Biosoft, Quebec, Canada) software. The *HMA3-D1* sequence of *Ae*. *tauschii* was amplified as two separate overlapping fragments using the primers TaHMA3-1 F (5’-TTGCTTGCAGCTTGTAGCTC-3’) and TaHMA3-1 R (5’-CATGTCGACGCTGAACTCCC-3’) for fragment 1 (1706-bp amplicon) and primers TaHMA3-2 F (5’-AGAGCAAGTCCAAGACGCAG-3’) and TaHMA3-2 R (5’-AGTCTCCTTTGTATTTTGCGCC-3’) for fragment 2 (1992-bp amplicon). PCR amplification was performed using a PTC-200 Thermocycler (MJ Research, Watertown, MA, USA). Each PCR was performed in a volume of 50 μL containing 200 ng of template DNA, 200 μmol/L of each dNTP, 100 μmol/L of each primer, 5.0 μL of 10 ×PCR buffer, 1 U Ex*Taq* DNA polymerase with high fidelity (TaKaRa, Dalian, China), and double-distilled (dd) H_2_O. The cycling conditions for fragment 1 amplification were initial denaturation at 95°C for 5 min, followed by 35 cycles of 94°C for 40 s, annealing at 58°C for 40 s, and extension at 72°C for 2 min, with a final extension at 72°C for 10 min. The cycling conditions for fragment 2 were initial denaturation at 95°C for 5 min followed by 35 cycles of 94°C for 40 s, annealing at 61°C for 40 s, and extension at 72°C for 2 min, with a final extension at 72°C for 10 min. The amplified products were separated on a 1.0% agarose gel in 1×TAE buffer (0.04 mol/L Tris base, 0.02 mol/L acetic acid, and 1.0 mmol/L EDTA) followed by staining with ethidium bromide. The PCR products were sequenced directly by Qingke (Chengdu, China).

### Sequence alignment and phylogenetic analysis

Multiple sequence alignments at both the nucleotide and predicted polypeptide levels were performed using DNAMAN v6.0 software (Lynnon Biosoft, Quebec, Canada). The transmembrane domains were predicted using TOPCONS (https://topcons.net/pred/result/rst_yGKZeW/). Phylogenetic trees were constructed based on the neighbor-joining method using MEGA v11 software [31]. Bootstrap analysis was based on 1,000 replicates.

## Results and discussion

### Results

#### *Ae*. *tauschii HMA3-D1*: Nucleotide sequence polymorphism

**Fig 1:**
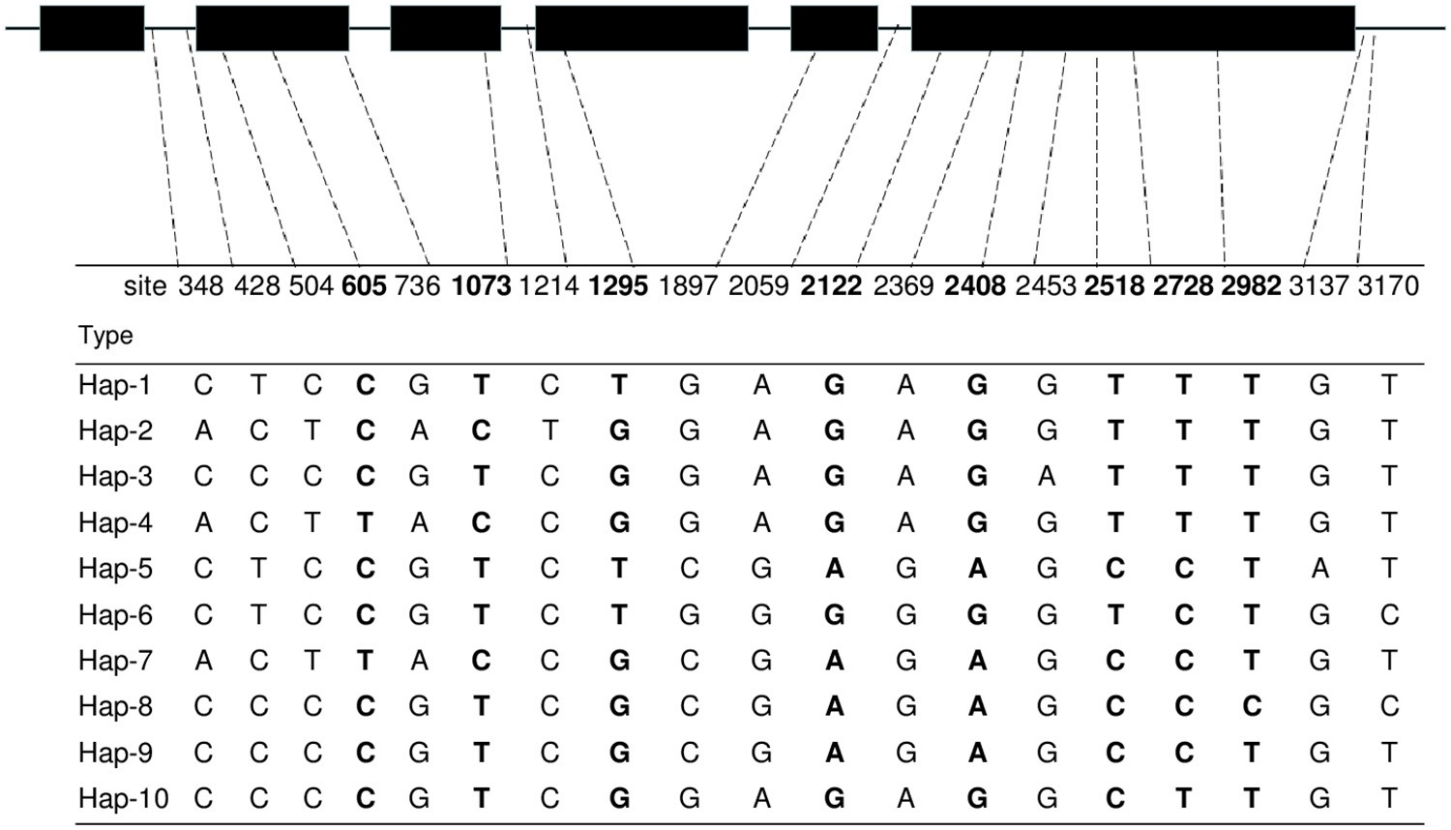
Haplotype variation of *HMA3-D1* in the 80 accessions of *Ae*. *tauschii*. Exons are depicted as a black box. Exonic polymorphisms generating a changed peptide are indicated in bold.

The full set of wheat *HMA3* homoeologous sequences from 80 *Ae*. *tauschii* accessions was deposited in GenBank with accession numbers OL739674 to OL739683 and revealed 10 haplotypes (Table 1). All homoeologs comprised six exons and five introns, compared with *TaHMA3-D1* (KF683298.1). The most common haplotype (Hap-4) was present in 18 accessions of ssp. *tauschii*, followed by Hap-1 (16 accessions) and Hap-10 (15 accessions). Both Hap-6 and Hap-8 were present in 8 accessions. Hap-2 and Hap-7 were each present in 4 accessions. Hap-3 and Hap-9 were present in 2 accessions. Finally, Hap-5 was present in 3 accessions. Exon variation involved thirteen single nucleotides, and eight of these thirteen polymorphisms induced an altered peptide sequence; the variation in the intron and downstream regions involved 4 single nucleotide substitutions and 2 polymorphisms, respectively (Fig 1).

In terms of geographical distribution (Table 1), most haplotypes were found in Turkey (8 haplotypes including Hap-1 to -3, -5 to -7, and -10) and Azerbaijan (8 haplotypes including Hap-1 to -2, -6 to -10), followed by Iran (6 haplotypes including Hap-1, -4 to -6, -8 and -10) and Afghanistan (5 haplotypes including Hap-2, -4, -7, -9 and -10). It is interesting that Turkey and Azerbaijan cover 9 of the ten haplotypes, except the most common haplotype (Hap-4).

### *Ae*. *tauschii HMA3-D1*: Peptide sequence polymorphism

**Fig 2:**
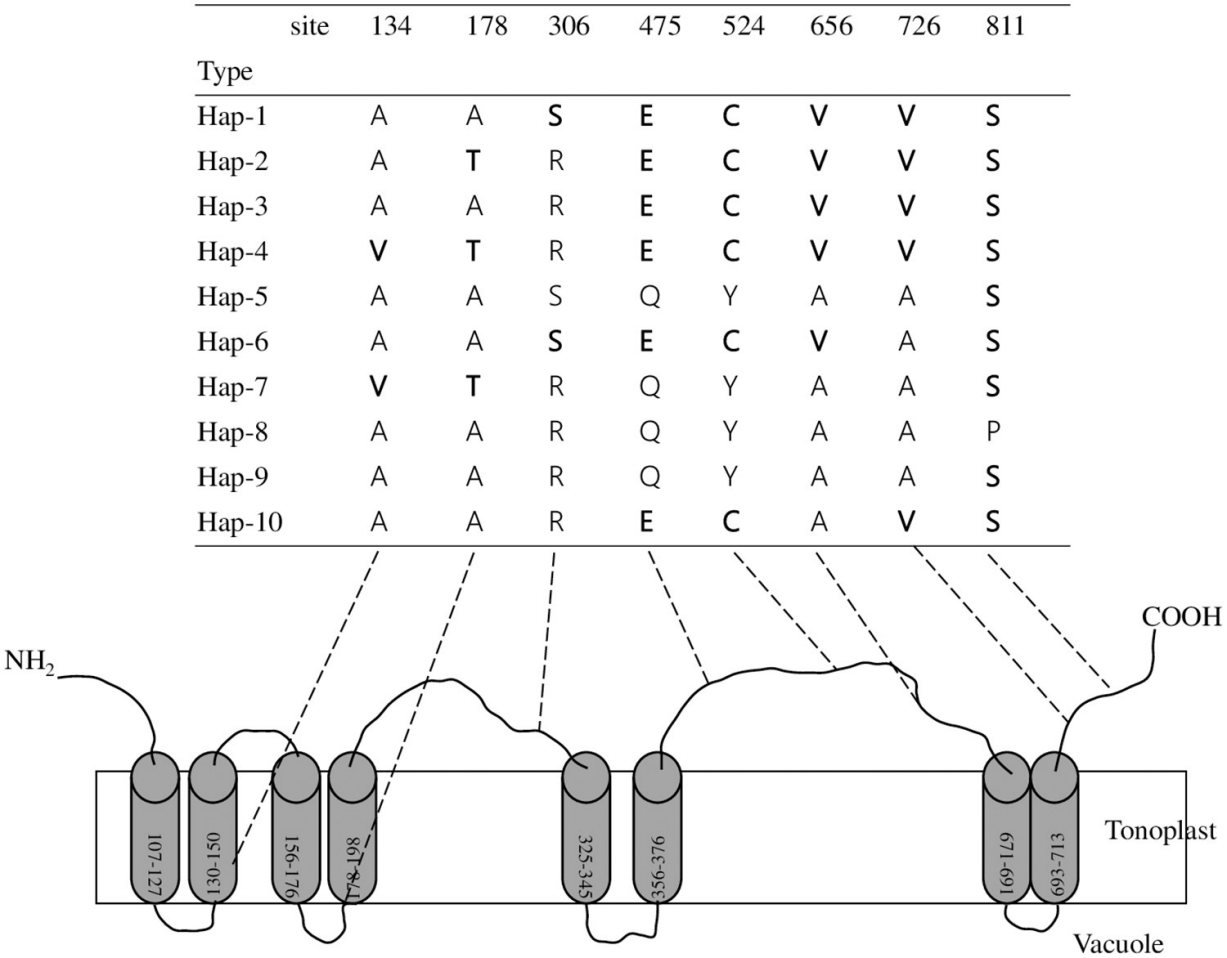
Polypeptide sequences of HMA3-D1 in the 80 accessions of *Ae*. *tauschii*. Polymorphisms generating a changed peptide are indicated in bold compared with Hap-8 (TaHMA3-D1). Transmembrane domains were predicted by TOPCONS.

At the polypeptide sequence level, the 10 haplotypes from *Ae*. *tauschii* collapsed into ten distinct polypeptides. Polypeptide sequence alignment of HMA3 homoeologs is shown in Fig 2. The predicted protein of *TaHMA3-D1* (AIA57682.1) in CS was indistinguishable from Hap-8. Their structural organization shared conserved transmembrane domains (TM1 to 8, boxed in Fig 2). Two of the eight altered amino acids were found in transmembrane domains. One was different from that of bread wheat, tetraploid wheat, barley, rice, maize, and *A*. *thaliana* at position 134, from A to V, in TM2, which was present in Hap-4 and -7 of *Ae*. *tauschii*, while the other was an A to T change at position 178 in TM4 in Hap-2, -4 and -7 (Fig 2). The remaining 6 altered peptide sequences were at position 306, from R to S, in Hap-1 and -6; position 475, from Q to E, and position 524, from Y to C, in Hap-1 to -4, -6 and -10; position 656, from A to V, in Hap-1 to -4 and -6; position 726, from A to V, in Hap-1 to -4 and -10; and position 811, from P to S, in Hap-1 to -10, except for Hap-9 (Fig 2).

### *HMA3-D1* homoeolog phylogeny

**Fig 3:**
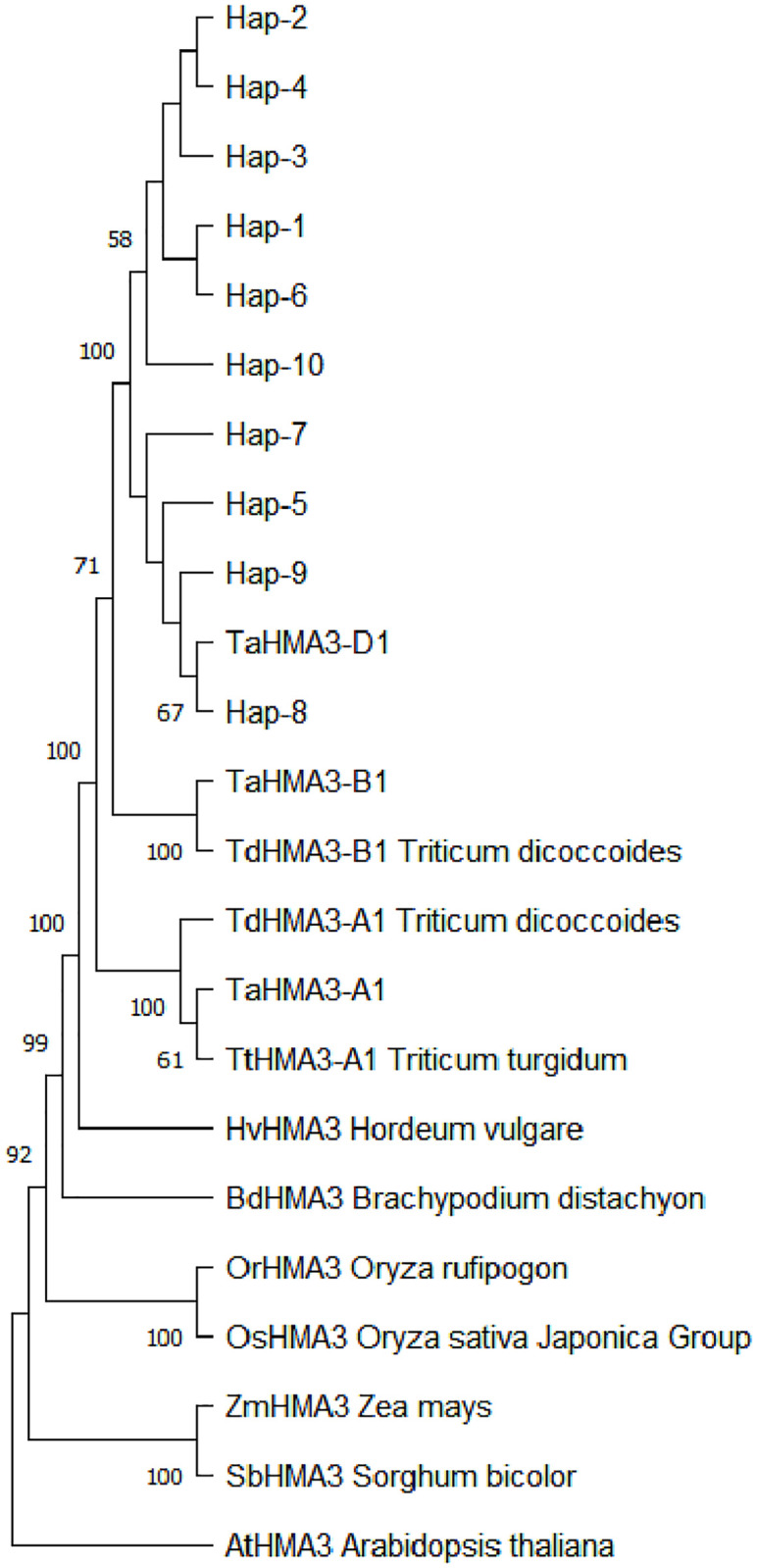
Phylogeny of *A*. *thaliana*, Brachypodium, rice, maize, barley, bread wheat cv. ‘Chinese Spring’, tetraploid wheat and *Ae*. *tauschii* HMA3 homoeologs obtained using the neighbor-joining method. Only bootstrap values (%) >50%, as calculated from 1,000 replicates, are shown.

The phylogeny of the protein sequences of wheat *HMA3* homoeologs is shown in Fig 3. Each of the three homoeologs from *Ae*. *tauschii* and *Triticum* formed a distinct clade. The homoeologs from the B and D genome clades appeared to be more closely related to one another than to the homoeologs from the A genome clade. Within the homoeologs from the D genome clade, two subgroups were recognizable: one comprised the *Ae*. *tauschii* sequences Hap-1 to -4, -6 and -10, and the other comprised the D genome sequences present in *Ae*. *tauschii* Hap-5, -7 to -9 and *T*. *aestivum*. The cluster of cereal sequences, including *Ae*. *tauschii*, *Triticum*, *Hordeum*, *Brachypodium*, *Oryza*, *Zea* and *Sorghum*, showed that they were phylogenetically closely related to one another. The *HMA3* homologous sequences of *Arabidopsis thaliana* (AT4G30120) as the outgroup.

### Discussion

Previous studies showed that *OsHMA3* from a low Cd-accumulating cultivar limits the translocation of Cd from the roots to the above ground tissues by selectively sequestrating Cd into the root vacuoles [5, 32]. Allelic variations in the coding sequences of this gene were responsible for the loss of function, such as a mutation at the 80th amino acid (R80H) [5], a mutation at the 380th amino acid in TM6 from Ser to Arg [7], a 153-bp (51-aa) deletion [6], and 14-aa deletion [8]. A strong association between the *BrHMA3* haplotypes and the Cd translocation phenotypes was also found, and variation in the *BrHMA3* coding sequence is a key determinant of Cd translocation to and accumulation in the leaves of *B*. *rapa* [33]. In durum wheat, the *TdHMA3-B1* (*Cdu-B1*) gene explained approximately 80% of the variation in grain Cd concentration [14–16]. The 17-bp duplication was responsible for the loss of function [16]. The Cd-associated SNPs identified in bread wheat are in a region of 5AL that is homoeologous to the region of *Cdu-B1* in durum [17].

The diverse *Ae*. *tauschii* offers a valuable gene pool for Cd tolerance [29]. Here, 80 resequenced *HMA3* homoeologs of *Ae*. *tauschii* were widely distributed and structured into six exons, and their sequences were highly homologous with one another (Fig 1). The sequence variation between them was concentrated more in the exonic than in intronic DNA (Fig 1). Ten haplotypes were revealed by these sequences and collapsed into ten distinct polypeptides (Fig 2). The structural organization of the *HMA3* homoeologs shared conserved transmembrane domains (TM1 to 8), where 6 of the eight altered amino acids were involved in the interval between transmembrane domains, and the remaining 2 were prospectively involved in TM1 and TM4. Furthermore, variation in the *BrHMA3* coding sequence is a key determinant of Cd translocation to and accumulation in the leaves of *B*. *rapa* [32]. Mutation at the 80th amino acid (R80H) in front of TM1 [5] and mutation in the 380th amino acid in TM6 from Ser to Arg [6] altered *OsHMA3* function (S1 Fig). Moreover, the bread wheat cultivars JiMai22 and HeNong6425 and the US wheat cultivar Fielder showed 3–7 times higher Cd translocation than the rice cv. Nipponbare. Overexpression of rice *OsHMA3* in wheat greatly decreases the Cd accumulation in grains [11]. Therefore, the altered peptide sequence revealed from *Ae*. *tauschii* has potential value for low/no Cd concentration wheat breeding.

*Ae*. *tauschii* has been taxonomically subdivided on the basis of its morphology into ssp. *tauschii* and ssp. *strangulata* [34–36]. It is widely accepted that ssp. *strangulata* is the source of the D genome in wheat [37–44]. Within the homoeologs from the D genome clade, two subgroups were recognizable: one comprised the *Ae*. *tauschii* sequences Hap-1 to -4, -6 and -10, and the other comprised the D genome sequences present in *Ae*. *tauschii* Hap-5, -7 to -9 and *T*. *aestivum* (Fig 3). The most common haplotype Hap-4, which has 7 altered amino acids compared to *TaHAM3-D1* from CS, was present only in *ssp*. *tauschii* (S1 Fig). Effective introduction and utilization of genetic resources present in *Ae*. *tauschii*, especially ssp. *tauschii* possessing high sequence variations, are a pressing need in wheat breeding and improvement.

## Supporting information

S1 Fig Polypeptide sequence alignment of *TaHMA3* homoeologs (*TaHMA3-D1*, *TaHMA3-B1* and *TaHMA3-A1*) from bread wheat cv. Chinese Spring (CS), *Ae*. *tauschii* haplotypes (Hap-1 to -10, given in the legends to Fig 1), *TdHMA3-B1a* (accession No. AIA57679.1) from *Triticum turgidum* subsp. Durum cultivar ‘8982-TL-L’, *HvHMA3* (accession No. AMK37440.1) from *Hordeum vulgare*, *ZmHMA3* (accession No. XP_020404933.1) from *Zea mays*, *OsHMA3* (accession No. XP_015647368.1) from *Oryza sativa* and *AtHMA3* (accession No. NP_194741.2) from *Arabidopsis thaliana*. Variations compared to *TaHMA3-D1* are indicated. Transmembrane domains (TM1-8) predicted by TOPCONS are shown in boxes.
(RTF)
