# Supplementary material for: Variation in the tonoplast cadmium transporter heavy metal ATPase 3 (HMA3) homolog gene in Aegilops tauschii
Source: PLoS One. 2023 Mar 3;18(3):e0279707. doi: 10.1371/journal.pone.0279707 (PMC9983875; doi:10.1371/journal.pone.0279707)
Supplement: S1 Fig — Variations compared to TaHMA3-D1 are indicated. Transmembrane domains (TM1-8) predicted by TOPCONS are shown in boxes. (RTF) [file pone.0279707.s001.rtf]

                           
TaHMA3-D1   MMGGGEPYTALEESLLSGEAAAR----------------RQ--WEKTYLDVLGVCCSAEVALVERLLAPLDGVRAVSVVVPSRTVVVEHDPAAVSQSRIV 82 
Hap-1       .......................----------------..--......................................................... 82 
Hap-2       .......................----------------..--......................................................... 82
Hap-3       .......................----------------..--......................................................... 82
Hap-4       .......................----------------..--......................................................... 82
Hap-5       .......................----------------..--......................................................... 82
Hap-6       .......................----------------..--......................................................... 82 
Hap-7       .......................----------------..--......................................................... 82
Hap-8       .......................----------------..--......................................................... 82 
Hap-9       .......................----------------..--.........................................................  82
Hap-10      .......................----------------..--......................................................... 82 
TaHMA3-A1  ......S.P...A...AE.....----------------..--.................................A....................... 82 
TaHMA3-B1  ......S.A.......PEQ....----------------..--......................................................... 82 
TdHMA3-B1a ......S.A.......PEQ....----------------..--......................................................... 82 
HvHMA3      .T.S..S.P...A....D....S----------------ARRK................................................S........ 84 
ZmHMA3      -----M.GP.V.AAARG.GCCGK----------------TAGK..........I..T............IN.....T........I.D..T.....FH.. 79 
OsHMA3      -.A.KDEAEG..AR..LLPPE.AAEEPTRCGGGDGGGGG.K--RK.............................V.....A............APE.A.. 97 
AtHMA3      ----------------MA.GEES----------------KKMNLQTS.F..V.I...S..SI.GNV.RQV...KEF..I......I.V..TFLI.PLQ.. 68

             
TM1                TM2                       TM3      TM4
TaHMA3-D1   KVLNGAGLEASVRAYGSSGVI-GRWPSPYIVACGALLLASSFRWLLPPLQWLALGAACAGAPPMLLRGLAAASRLTLDINILMLIAVAGAVALKDYAEAG 181  
Hap-1       .....................-.............................................................................. 181
Hap-2       .....................-..........................................................................T... 181
Hap-3       .....................-.............................................................................. 181
Hap-4       .....................-..............................V...........................................T... 181
Hap-5       .....................-.............................................................................. 181
Hap-6       .....................-.............................................................................. 181
Hap-7       .....................-..............................V...........................................T... 181
Hap-8       .....................-.............................................................................. 181
Hap-9       .....................-.............................................................................. 181
Hap-10      .....................-.............................................................................. 181 
TaHMA3-A1   ...................F.-..R.....................L.................V..........A....................T... 181
TaHMA3-B1   .....................-............V...................A.............I...........................T... 181
TdHMA3-B1a  .....................-............V...................A.............I...........................T... 181
HvHMA3      .....................-..........................................V...F......A...........V........T... 183
ZmHMA3      ....K.............AGAP......F.....V..A..L.AP.....R...VA...V.SQ.....AF...GK...................GS.T... 179
OsHMA3      .A..K...............V-S.........S.V..T..F.E..F....C..VA.VV......VR..F......S....V.........LC.G..T... 196
AtHMA3      .A..Q.R......P..ETSLK-SQ....FAIVS.V..VL.F.KYFYS..E...IV.VV..VF.I.AKAV.SVT.FR....A.T....IATLCMQ.FT..A 167


TM4
TaHMA3-D1  VIVFLFTTAEWLETLACTKASAGMSSLMSMIPPKAVLAETGEVVNVRDIGVGAVIAVRAGEMVPVDGVVVDGQSEVDERSLTGESYPVPKQPQSEVWAGT 281
Hap-1       .................................................................................................... 281
Hap-2       .................................................................................................... 281
Hap-3       .................................................................................................... 281
Hap-4       .................................................................................................... 281
Hap-5       .................................................................................................... 281
Hap-6       .................................................................................................... 281
Hap-7       .................................................................................................... 281
Hap-8       .................................................................................................... 281
Hap-9       .................................................................................................... 281
Hap-10      .................................................................................................... 281
TaHMA3-A1   ....................................................V............................................... 281
TaHMA3-B1   ...................................................................M........................H....... 281
TdHMA3-B1a  .........................................D.........................M........................H....... 281
HvHMA3      .................................................D..........................................L....... 283
ZmHMA3      A......V................L....SV.KTV......Q..GMG.VA..T.V.....DV........G.......S......F.....A........ 279
OsHMA3      A...........................G.L.V...I.T.....S...VR..D.V......I.......................F......H....... 296 
AtHMA3      T.....SV.D...SS.AH...IV......LA.R...I.D..LE.D.DEV.INT.VS.K...SI.I.......SCD...KT.....F..S..RE.T.M.A. 267 


TM5                        TM6
TaHMA3-D1   LNLDGYIAVRTMALAENSTVAKMERLVEEAQQSKSKTQRLIDSCARYYTPAVVVLGAGVALLPPLLGARDAERWFRLALVLLVSACPCALVLSTPVATFC 381
Hap-1       ........................S........................................................................... 381
Hap-2       .................................................................................................... 381
Hap-3       .................................................................................................... 381 
Hap-4       .................................................................................................... 381 
Hap-5       ........................S...........................................................................381 
Hap-6       ........................S........................................................................... 381
Hap-7       .................................................................................................... 381
Hap-8       .................................................................................................... 381 
Hap-9       .................................................................................................... 381 
Hap-10      .................................................................................................... 381
TaHMA3-A1   .............................................K...................................................... 381
TaHMA3-B1   ...................................R.........K...................................................... 381
TdHMA3-B1a  ...................................R.........K...................................................... 381
HvHMA3      ...........S.................................K.......F...........V.................................. 383
ZmHMA3      I......S...T...................N.R.R.........KH........A...V.V.V....P.L.H....S...................... 379
OsHMA3      M.F........T................A..N.R...........K........VA.....I.A....DGL.Q.WK....M................S.. 396
AtHMA3      I..N...K.K.T...RDCV....TK......K.QT....F..K.S.........SA.CF.VI.V..KVQ.LSH..H....V...G...G.I......... 367


TaHMA3-D1   ALLTAARMGVLVKGGDVLESLGEIRAVAFDKTGTITRGEFSVDMFDVVGHKVQ-MSYLLYWISSIESKSSHPMAAALVEYAQSKSIEPKPECVAQFRILP 480
Hap-1       .....................................................-........................................E..... 480
Hap-2       .....................................................-........................................E..... 480
Hap-3       .....................................................-........................................E..... 480
Hap-4       .....................................................-........................................E..... 480
Hap-5       .....................................................-.............................................. 480
Hap-6       .....................................................-........................................E..... 480
Hap-7       .....................................................-.............................................. 480
Hap-8       .....................................................-.............................................. 480
Hap-9       .....................................................-.............................................. 480
Hap-10      .....................................................-........................................E..... 480
TaHMA3-A1   ........................................T.......EQ...-..H......................H......Q.......E..... 480
TaHMA3-B1   ........................K...............T............-..H......................H..............E..V.. 480
TdHMA3-B1a  ........................K...............T............-..H......................H.R............E..V.. 480
HvHMA3      .........L..............K...............T..I.........-..Q.....................................E..... 482
ZmHMA3      ...R.....L.....N.......V.VA..............IKD.L..RD...-..Q....V........................Q....D.TETC.YH 478 
OsHMA3      .M.R.....IFI.............................I.S.HL..DHKVE.DH.....A.......................Q.N..N.GD...Y. 496
AtHMA3      ..TK..TS.F.I.T..C..T.AK.KI..........KA..M.SD.RSLSPSIN-LHK....V....C..........ID..R.V.V....DI.EN.QNF. 466 

      


       

TaHMA3-D1   GEGIYGEIDGKRIYVGNKRVLARGSSC-QTVPERMNGLKGVSIGYVICDGDLVGVFSFSDDCRTGAAEAIRELASMGISSVLLTGDSAEAAVHAQQQLGG 579
Hap-1       ...........................-................C....................................................... 579 
Hap-2       ...........................-................C....................................................... 579 
Hap-3       ...........................-................C....................................................... 579
Hap-4       ...........................-................C....................................................... 579
Hap-5       ...........................-........................................................................ 579
Hap-6       ...........................-................C....................................................... 579
Hap-7       ...........................-........................................................................ 579
Hap-8       ...........................-........................................................................ 579 
Hap-9       ...........................-........................................................................ 579
Hap-10      ...........................-................C....................................................... 579
TaHMA3-A1   ...V.......................-.............................L.....................................ER... 579 
TaHMA3-B1   ..........M................-..............M..............L............................I....M...E.... 580
TdHMA3-B1a  ..........M................-..............M..............L............................I....M...E.... 580
HvHMA3      .......................A...-..AVPERMNGLKGVSIGYVICDGDLVGVFSLS.DCRTG.AEAIRELASMGI.SV.LTGDSAE.AVHA.ERL. 582
ZmHMA3      ......A.N..H..I..E.IM..S.CRQ.EAGH.ETDGLKGVSVGLVICDGDLVGKFSLS.TCRTG.AEAILQLRSMGIKSVMLTGDS...AKHA.EQL. 578 
OsHMA3      ........H..H..I..R.T...A..P-.STQ.MGEMI............E.A....L...............G.L..K..M.....SA..T...G.... 595
AtHMA3      ...V..R...QD..I....IAQ.AGCLTDN..DIEATM.RGKTIGY.YM.AKLTGSFNLL.GCRYGVAQALKELKS------------------------ 542 


TM7
TaHMA3-D1   ALEEL-HSELFPEDKVRLVG-ALKARAGPTMMVGDGMNDAPALATADVGVSMGISGSAAAMETSHATLMSSDILRVPEAVRLGRRARRTIAVNMVSSIAA 677
Hap-1       .....-..............-.........................................................V..................... 677
Hap-2       .....-..............-.........................................................V..................... 677
Hap-3       .....-..............-.........................................................V..................... 677
Hap-4       .....-..............-.........................................................V..................... 677
Hap-5       .....-..............-............................................................................... 677
Hap-6       .....-..............-.........................................................V..................... 677
Hap-7       .....-..............-............................................................................... 677
Hap-8       .....-..............-............................................................................... 677
Hap-9       .....-..............-............................................................................... 677
Hap-10      .....-..............-............................................................................... 677
TaHMA3-A1   .....-..............-............................................................................V.. 677 
TaHMA3-B1   ..L.EV..............-.........................................................................A..V.. 678
TdHMA3-B1a  ..L.EV..............-.........................................................................A..V.. 678
HvHMA3      GAL.E-LHSELFPEDKVRLVS.V...V.................M....................................................V.. 680 
ZmHMA3      GVL.E-LHSGLLPEDKVRLIRG.Q..H.A.L.............A........L......I.........G.V....K.......T.......VA..VG. 677 
OsHMA3      VM...-....L........S-G....F.............A...A...........................V...........C........VAG.V.V 693
AtHMA3      ---------------------------------------------------------------------------------------------------- 542 


TM7         TM8
TaHMA3-D1  KAAVLALAVAWRPVLWAAVLADVGTCLLVVLNSMLLLGEGRGRRGKEE------------------------ACRATARSLEMRRSQLAAVSPDAAAKSV 753
Hap-1       ................................................------------------------V........................... 753 
Hap-2       ................................................------------------------V........................... 753
Hap-3       ................................................------------------------V........................... 753
Hap-4       ................................................------------------------V........................... 753
Hap-5       ................................................------------------------............................ 753
Hap-6       ................................................------------------------............................ 753
Hap-7       ................................................------------------------............................ 753
Hap-8       ................................................------------------------............................ 753
Hap-9       ................................................------------------------............................ 753
Hap-10      ................................................------------------------V........................... 753
TaHMA3-A1  .........................................R......------------------------........................T... 753
TaHMA3-B1  .......................................R.......D------------------------....................S....TKS 754
TdHMA3-B1a .......................................R.......D------------------------....................S.S..TKS 754
HvHMA3      .V......L...............................G.......------------------------........................T... 756 
ZmHMA3      ................V..............H.....RDAARA.RRCGGASKACCATACKAPKTACCATASK..G..TVKPVAT.P.P.GAGKK.DRPGG 777 
OsHMA3      ........A.........................T..R.EWKGGA..DG-----------------------.........V.-......D.QAPN.ADA 769 
AtHMA3      ---------------------------------------------------------------------------------------------------- 542

     


TaHMA3-D1   GKTGGDASKGCHCCHKPSK-SPEHSVAIDVRAGEQRESPTAATCAPAKKVEVTGSVNGPAAPASSSGASVGCCPRETDSTEACKKMAPADLVLNICTTFG 852
Hap-1        ...................-......................................S......................................... 852
Hap-2        ...................-......................................S......................................... 852
Hap-3        ...................-......................................S......................................... 852
Hap-4        ...................-......................................S......................................... 852 
Hap-5       ...................-......................................S......................................... 852
Hap-6        ...................-......................................S......................................... 852
Hap-7        ...................-......................................S......................................... 852
Hap-8        ...................-................................................................................ 852
Hap-9        ...................-......................................S......................................... 852
Hap-10       ...................-......................................S......................................... 852
TaHMA3-A1   .......P..........R-...........VD....G.............Y-----------...CV.A...SP------------------------- 816
TaHMA3-B1   VGKT.GDASKGCH.CHKPSR............D....G...................ASVM.....CV.A...SP------------------------- 829
TdHMA3-B1a  VGKT.GDASKGCH.CHKPSR............D....G...................ASVM.....CV.A...SP------------------------- 829
HvHMA3      .................I.-......V.N..VD....G..D...T...N.....L.DA-----.VMP..SS.VSGGGCCSREKTGRNM------------ 836
ZmHMA3      D.H.NGKDDCHR....Q..-L..DA.V.AIPVRAVEHRKD..AHEK.EGNAAG.CCG.-----APAS.ACCAEAHGGEDEVCIVIS.RSPCCSTARSRSA 871
OsHMA3      .AA.REQTN..R..P..GM-......V..I..DGE.QEERP.EA.VVA.CCGG.GGE.IRCG..--------------------.KPT.TV.VAK.CGG.848 
AtHMA3      ---------------------------------------------------------------------------------------------------- 542


TaHMA3-D1   L--------------------------------------------------------------------------------------------------- 853
Hap-1       .--------------------------------------------------------------------------------------------------- 853
Hap-2       .--------------------------------------------------------------------------------------------------- 853
Hap-3       .--------------------------------------------------------------------------------------------------- 853
Hap-4       .--------------------------------------------------------------------------------------------------- 853
Hap-5       .--------------------------------------------------------------------------------------------------- 853
Hap-6       .--------------------------------------------------------------------------------------------------- 853
Hap-7       .--------------------------------------------------------------------------------------------------- 853
Hap-8       .--------------------------------------------------------------------------------------------------- 853
Hap-9       .--------------------------------------------------------------------------------------------------- 853
Hap-10      .--------------------------------------------------------------------------------------------------- 853
TaHMA3-A1   ---------------------------------------------------------------------------------------------------- 816
TaHMA3-B1   ---------------------------------------------------------------------------------------------------- 829
TdHMA3-B1a  ---------------------------------------------------------------------------------------------------- 829
HvHMA3      ---------------------------------------------------------------------------------------------------- 838
ZmHMA3      SPKDAMCCGSGGKDGGAISALVC----------------------------------------------------------------------------- 894
OsHMA3      GGGEGTRCGASKNPATAAVVAKCCSGGGGEGIGCGASKKPTATAVVAKCCGGGGEGTRCAASKKPATAAVVAKCCGGDGGEGTGCGASKRSPPAEGSCSG 948 
AtHMA3      ---------------------------------------------------------------------------------------------------- 542


                

TaHMA3-D1    -------------------------------------------------------- 853
Hap-1        -------------------------------------------------------- 853
Hap-2        -------------------------------------------------------- 853
Hap-3        -------------------------------------------------------- 853
Hap-4        -------------------------------------------------------- 853
Hap-5        -------------------------------------------------------- 853
Hap-6        -------------------------------------------------------- 853
Hap-7        -------------------------------------------------------- 853
Hap-8        -------------------------------------------------------- 853
Hap-9        -------------------------------------------------------- 853
Hap-10       -------------------------------------------------------- 853
TaHMA3-A1   -------------------------------------------------------- 816
TaHMA3-B1   -------------------------------------------------------- 829
TdHMA3-B1a  -------------------------------------------------------- 829
HvHMA3       -------------------------------------------------------- 838
ZmHMA3       -------------------------------------------------------- 894
OsHMA3       GEGGTNGVGRCCTSVKRPTCCDMGAAEVSDSSPETAKDCRNGRCCAKTMNSGEVKG 1004
AtHMA3       -------------------------------------------------------- 542


S1 Fig. Polypeptide sequence alignment of TaHMA3 homoeologs (TaHMA3-D1, TaHMA3-B1 and TaHMA3-A1) from bread wheat cv. Chinese Spring (CS), Ae. tauschii haplotypes (Hap-1 to -10, given in the legends to Fig. 1), TdHMA3-B1a (accession No. AIA57679.1) from Triticum turgidum subsp. Durum cultivar ‘8982-TL-L’, HvHMA3 (accession No. AMK37440.1) from Hordeum vulgare, ZmHMA3 (accession No. XP_020404933.1) from Zea mays, OsHMA3 (accession No. XP_015647368.1) from Oryza sativa and AtHMA3 (accession No. NP_194741.2) from Arabidopsis thaliana. Variations compared to TaHMA3-D1 are indicated. Transmembrane domains (TM1-8) predicted by TOPCONS are shown in boxes. 
